# UbiHub: a data hub for the explorers of ubiquitination pathways

**DOI:** 10.1093/bioinformatics/bty1067

**Published:** 2019-01-02

**Authors:** Lihua Liu, David R Damerell, Leonidas Koukouflis, Yufeng Tong, Brian D Marsden, Matthieu Schapira

**Affiliations:** bty1067-aff1 Structural Genomics Consortium, University of Toronto, Toronto, ON, Canada; bty1067-aff2 Structural Genomics Consortium, University of Oxford, Headington Oxford, Oxfordshire, UK; bty1067-aff3 Department of Pharmacology and Toxicology, University of Toronto, Toronto, ON, Canada; bty1067-aff4 Department of Chemistry and Biochemistry, University of Windsor, Windsor, ON, Canada; bty1067-aff5 Kennedy Institute of Rheumatology, Nuffield Department of Orthopaedics, Rheumatology and Musculoskeletal Sciences, University of Oxford, Roosevelt Drive, Headington, Oxford, Oxfordshire, UK

## Abstract

**Motivation:**

Protein ubiquitination plays a central role in important cellular machineries such as protein degradation or chromatin-mediated signaling. With the recent discovery of the first potent ubiquitin-specific protease inhibitors, and the maturation of proteolysis targeting chimeras as promising chemical tools to exploit the ubiquitin-proteasome system, protein target classes associated with ubiquitination pathways are becoming the focus of intense drug-discovery efforts.

**Results:**

We have developed UbiHub, an online resource that can be used to visualize a diverse array of biological, structural and chemical data on phylogenetic trees of human protein families involved in ubiquitination signaling, including E3 ligases and deubiquitinases. This interface can inform target prioritization and drug design, and serves as a navigation tool for medicinal chemists, structural and cell biologists exploring ubiquitination pathways.

**Availability and implementation:**

https://ubihub.thesgc.org.

## 1 Introduction

In the past 2 years, important progress was made in drug-discovery targeting ubiquitination pathways. First, potent, selective and reversible chemical inhibitors of ubiquitin-specific proteases (USPs), a protein family that had resisted intense medicinal chemistry efforts for over a decade, were discovered (Gavory *et al.*, 2017; Kategaya *et al.*, 2017; Liang *et al.*, 2014; Turnbull *et al.*, 2017). Second, proteolysis targeting chimeras (PROTACs)—heterobifunctional molecules that recruit E3 ligases to protein targets for ubiquitination and subsequent proteasomal degradation—matured from a novel chemical biology concept to a promising paradigm for drug discovery (Churcher, 2018). To date, selective inhibitors were disclosed for 2 out of 57 USPs in the human genome (USP1 and USP7) (Gavory *et al.*, 2017; Kategaya *et al.*, 2017; Liang *et al.*, 2014; Turnbull *et al.*, 2017), and PROTACs are currently exploiting 5 out of > 600 human E3 ligases (VHL, CRBN, MDM2, IAPs and DCAF15) (Chan *et al.*, 2018; Demizu *et al.*, 2016; Fischer *et al.*, 2014; Han *et al.*, 2017; Ohoka *et al.*, 2017; Schneekloth *et al.*, 2008; Testa *et al.*, 2018; Uehara *et al.*, 2017). The rapidly growing body of data on the biology, structure and chemistry of these emerging and important target classes will guide target selection and drug design.

Here, we present UbiHub, an online data hub where drug-discovery scientists focused on ubiquitination pathways can easily navigate data of relevance to their work. The UbiHub graphic user interface is based on the representation of protein families as phylogenetic trees, onto which heterogeneous data collected from diverse repositories and the literature can be projected and scrutinized.

## 2 Materials and methods

### 2.1 Assembling protein families

Four protein families are included in UbiHub: 8 E1 ubiquitin activating enzymes, 41 E2 ubiquitin conjugating enzymes, 634 E3 ubiquitin ligases and 113 de-ubiquitinases (DUBs). The composition of each family was derived from searches of their respective signature domains in the PFAM (Finn *et al.*, 2014), and SMART (Schultz *et al.*, 2000) databases. Previously reported atypical enzymes were added to the E1 list (Schulman and Harper, 2009). The E3 ligases list was complemented with a previously reported genome-wide functional annotation of human E3s and a systematic inventory of DCAFs (Lee and Zhou, 2007; Li *et al.*, 2008). To improve visibility of the very large E3 family, it was divided into 297 proteins relying on multi-subunit complexes (mostly E3s interacting with Cullins, adaptor proteins and E2-recruiting subunits) and 337 standalone E3 ligases. DUBs were divided into 57 USPs and 56 functionally related, but biochemically distinct non-USP proteins. The composition and subfamily classification of DUBs was based on a previously reported inventory of deubiquitinating enzymes (Nijman *et al.*, 2005) and on the latest developments in the field (Kwasna *et al.*, 2018; Maurer and Wertz, 2016).

### 2.2 Ubiquitin-proteasome system association

Ubiquitination can serve as a signal for ubiquitin-proteasome system (UPS)-mediated degradation or other non-degradation related signaling pathways. The association of E3 ligases to the UPS was estimated automatically and assigned a confidence score of 0 (no indication of UPS association) to 3 (reliable UPS association) based on 3 criteria. First we looked whether the word ‘degrad’ was found in the Function section of the UniProt entry of the protein (UniProt Consortium, 2018). Second, we searched for the word ‘degrad’ among the Reactome pathways (Fabregat *et al.*, 2018) linked to the protein. Third, we compiled for each E3 ligase the list of Reactome pathways assigned to all protein interactors from the BioGrid database (Stark, 2006), and searched for the word ‘degrad’ in the pathways that were enriched among these interactors (pathways enriched at least three times compared with their prevalence in the human proteome, and found in at least three interactors). The UPS association score was set to 0, 1, 2 or 3 when none, one, two or all of these conditions were met respectively. Upon literature review of over 30 random E3s, we found the score to be reasonable in over 90% of cases, and adjusted it manually when it was found inaccurate.

### 2.3 Phylogenetic trees and data collection

Phylogenetic trees are generated, and biological, structural and chemical data collected as previously described for ChromoHub (Liu *et al.*, 2012; Shah *et al.*, 2014), and stored in a MySQL database. Additionally, gene essentiality in cancer is extracted from the Broad Institute’s cancer dependency map, where we use data from CRISPR-knockout studies and essentiality scores corrected for copy-number effect, and data from RNAi knock-down studies using DEMETER2 normalization (McFarland *et al.*, 2018; Meyers *et al.*, 2017).

## 3 Results

The graphical user interface is based on zoom-able phylogenetic trees that represent any pre-selected protein family. In the case of E3 ligases, users can choose to only display proteins that are associated with the UPS with a pre-defined confidence level. A checkbox menu allows users to simultaneously tag proteins on a tree with diverse icons related to biological, structural or chemical data. Clicking on any of these icons brings pop-up windows with figures providing further details and html links to the source of information (PubMed record or public repository such as PDB entry). The checkbox menu includes click-able ‘?’ symbols next to each menu item that can be used to display information on the data source and the way the data were processed. Through this graphical interface, users can have a bird’s-eye view of the disease association landscape of an entire protein family, medicinal chemists can rapidly retrieve compounds co-crystallized with their protein target, structural biologists can inspect the structural coverage of a protein or its phylogenetic neighbors, and cell biologists can find the *K_D_* or IC_50_ and selectivity profile of chemical inhibitors, produce the chemical coverage of E3 ligases involved in the UPS, or quickly visualize the cancer dependency map of USPs.

## References

[bty1067-B1] Chan K.H. et al (2018) Impact of Target Warhead and Linkage Vector on Inducing Protein Degradation: comparison of Bromodomain and Extra-Terminal (BET) Degraders Derived from Triazolodiazepine (JQ1) and Tetrahydroquinoline (I-BET726) BET Inhibitor Scaffolds. J. Med. Chem., 61, 504–513. 28595007 10.1021/acs.jmedchem.6b01912 PMC5788402

[bty1067-B2] Churcher I. (2018) Protac-induced protein degradation in drug discovery: breaking the rules or just making new ones? J. Med. Chem., 61, 444–452. 29144739 10.1021/acs.jmedchem.7b01272

[bty1067-B3] Demizu Y. et al (2016) Development of BCR-ABL degradation inducers via the conjugation of an imatinib derivative and a cIAP1 ligand. Bioorganic Med. Chem. Lett., 26, 4865–4869. 10.1016/j.bmcl.2016.09.041 27666635

[bty1067-B4] Fabregat A. et al (2018) The reactome pathway knowledgebase. Nucleic Acids Res., 46, D649–D655. 29145629 10.1093/nar/gkx1132 PMC5753187

[bty1067-B5] Finn R.D. et al (2014) PFAM: the protein families database. Nucleic Acids Res., 42, D222–D230. 24288371 10.1093/nar/gkt1223 PMC3965110

[bty1067-B6] Fischer E.S. et al (2014) Structure of the DDB1-CRBN E3 ubiquitin ligase in complex with thalidomide. Nature, 512, 49–53. 25043012 10.1038/nature13527 PMC4423819

[bty1067-B7] Gavory G. et al (2018) Discovery and characterization of highly potent and selective allosteric USP7 inhibitors. Nat. Chem. Biol., 14, 118–125. 29200206 10.1038/nchembio.2528

[bty1067-B8] Han T. et al (2017) Anticancer sulfonamides target splicing by inducing RBM39 degradation via recruitment to DCAF15. Science, 356, eaal3755. 28302793 10.1126/science.aal3755

[bty1067-B9] Kategaya L. et al (2017) USP7 small-molecule inhibitors interfere with ubiquitin binding. Nature, 550, 534–538. 29045385 10.1038/nature24006

[bty1067-B10] Kwasna D. et al (2018) Discovery and characterization of ZUFSP/ZUP1, a distinct deubiquitinase class important for genome stability. Mol. Cell, 70, 150–164. 29576527 10.1016/j.molcel.2018.02.023 PMC5896202

[bty1067-B11] Lee J., Zhou P. (2007) DCAFs, the missing link of the CUL4-DDB1 ubiquitin ligase. Mol. Cell, 26, 775–780. 17588513 10.1016/j.molcel.2007.06.001

[bty1067-B12] Li W. et al (2008) Genome-wide and functional annotation of human E3 ubiquitin ligases identifies MULAN, a mitochondrial E3 that regulates the organelle’s dynamics and signaling. PLoS One, 3, e1487. 18213395 10.1371/journal.pone.0001487 PMC2198940

[bty1067-B13] Liang Q. et al (2014) A selective USP1-UAF1 inhibitor links deubiquitination to DNA damage responses. Nat. Chem. Biol., 10, 298–304. 24531842 10.1038/nchembio.1455 PMC4144829

[bty1067-B14] Liu L. et al (2012) ChromoHub: a data hub for navigators of chromatin-mediated signalling. Bioinformatics, 28, 2205–2206. 22718786 10.1093/bioinformatics/bts340 PMC3413389

[bty1067-B15] Maurer T., Wertz I.E. (2016) Length matters: MINDY is a new deubiquitinase family that preferentially cleaves long polyubiquitin chains. Mol. Cell, 63, 4–6. 27392144 10.1016/j.molcel.2016.06.027

[bty1067-B16] McFarland J.M. et al (2018) Improved estimation of cancer dependencies from large-scale RNAi screens using model-based normalization and data integration. Nat. Commun., 9, 4610. 30389920 10.1038/s41467-018-06916-5 PMC6214982

[bty1067-B17] Meyers R.M. et al (2017) Computational correction of copy number effect improves specificity of CRISPR-Cas9 essentiality screens in cancer cells. Nat. Genet., 49, 1779–1784. 29083409 10.1038/ng.3984 PMC5709193

[bty1067-B18] Nijman S.M.B. et al (2005) A genomic and functional inventory of deubiquitinating enzymes. Cell, 123, 773–786. 16325574 10.1016/j.cell.2005.11.007

[bty1067-B19] Ohoka N. et al (2017) In vivo knockdown of pathogenic proteins via specific and nongenetic Inhibitor of Apoptosis Protein (IAP)-dependent protein erasers (SNIPERs). J. Biol. Chem., 292, 4556–4570. 28154167 10.1074/jbc.M116.768853 PMC5377772

[bty1067-B20] Schneekloth A.R. et al (2008) Targeted intracellular protein degradation induced by a small molecule: en route to chemical proteomics. Bioorganic Med. Chem. Lett., 18, 5904–5908. 10.1016/j.bmcl.2008.07.114 PMC3175619 18752944

[bty1067-B21] Schulman B.A., Harper J.W. (2009) Ubiquitin-like protein activation by E1 enzymes: the apex for downstream signalling pathways. Nat. Rev. Mol. Cell Biol., 10, 319–331. 19352404 10.1038/nrm2673 PMC2712597

[bty1067-B22] Schultz J. et al (2000) SMART: a web-based tool for the study of genetically mobile domains. Nucleic Acids Res., 28, 231–234. 10592234 10.1093/nar/28.1.231 PMC102444

[bty1067-B23] Shah M.A. et al (2014) ChromoHub V2: cancer genomics. Bioinformatics, 30, 590–592. 24319001 10.1093/bioinformatics/btt710 PMC3928521

[bty1067-B24] Stark C. et al (2006) BioGRID: a general repository for interaction datasets. Nucleic Acids Res., 34, D535–D539. 16381927 10.1093/nar/gkj109 PMC1347471

[bty1067-B25] Testa A. et al (2018) 3-Fluoro-4-hydroxyprolines: synthesis, conformational analysis, and stereoselective recognition by the VHL E3 ubiquitin ligase for targeted protein degradation. J. Am. Chem. Soc., 140, 9299–9313. 29949369 10.1021/jacs.8b05807 PMC6430500

[bty1067-B26] Turnbull A.P. et al (2017) Molecular basis of USP7 inhibition by selective small-molecule inhibitors. Nature, 550, 481–486. 29045389 10.1038/nature24451 PMC6029662

[bty1067-B27] Uehara T. et al (2017) Selective degradation of splicing factor CAPERα By anticancer sulfonamides. Nat. Chem. Biol., 13, 675–680. 28437394 10.1038/nchembio.2363

[bty1067-B30] UniProt Consortium, T. (2018) UniProt: the universal protein knowledgebase. Nucleic Acids Res., 46, 2699. 29425356 10.1093/nar/gky092 PMC5861450

